# Comparison of Alterations in miRNA Expression in Matched Tissue and Blood Samples during Spinal Cord Glioma Progression

**DOI:** 10.1038/s41598-019-42364-x

**Published:** 2019-06-24

**Authors:** Tian An, Tao Fan, Xin Qing Zhang, Yu-Fei Liu, Jiangpinghao Huang, Cong Liang, Bo-Han Lv, Yin-Qian Wang, Xin-Gang Zhao, Jia-Xian Liu, Yu- Huan Fu, Guang-Jian Jiang

**Affiliations:** 1 Traditional Chinese Medicine School, Beijing University of Chinese Medicine, Beijing, 100029 China; 2 Department of Neurosurgery, Sanbo Brain Hospital, Capital Medical University, Beijing, 100093 China; 3 Department of Neurosurgery, ChuiYangLiu Hospital affiliated to Tsinghua University, Beijing, 100022 China; 4 The Third Affiliated Hospital, Beijing University of Chinese Medicine, Beijing, 100029 China; 5 University of Southern California, Los Angeles, CA 90007 USA; 6 Molecular Development and Diagnosis of Tumor Pathology, Department of Basic Medicine, Tangshan Vocational and Technical College, Tangshan, 063000 China

**Keywords:** Cancer genomics, Prognostic markers, Tumour biomarkers

## Abstract

Abnormal expression of microRNAs (miRNAs) contributes to glioma initiation. However, the expression of miRNAs in tumour tissue or blood of spinal cord glioma (SCG) patients, particularly in high-grade spinal gliomas (Grade IV) known as glioblastoma (GBM), remains largely unknown. In this study we aimed to determine differentially expressed miRNAs (DEmiRNAs) in the tissue and blood between spinal cord glioblastoma (SC-GBM) patients and low grade SCG (L-SCG) patients. Additionally, we predicted key miRNA targets and pathways that may be critical in glioma development using pathway and gene ontology analysis. A total of 74 miRNAs were determined to be differentially expressed (25 upregulated and 49 downregulated) in blood, while 207 miRNAs (20 up-regulated and 187 down-regulated) were identified in tissue samples. Gene ontology analysis revealed multicellular organism development and positive regulation of macromolecule metabolic process to be primarily involved. Pathway analysis revealed “Glioma”, “Signalling pathways regulating pluripotency of stem cells” to be the most relevant pathways. miRNA-mRNA analysis revealed that hsa-miRNA3196, hsa-miR-27a-3p, and hsa-miR-3664-3p and their target genes are involved in cancer progression. Our study provides a molecular basis for SCG pathological grading based on differential miRNA expression.

## Introduction

Glioma is one of the most common and aggressive primary malignant tumours, whether in the spinal cord or in the brain, of which Spinal cord gliomas (SCG) account for more than 80% of spinal cord tumours^1^. Spinal cord glioma is difficult to be totally resected; postoperative radiotherapy and chemotherapy are both uncertain; comprehensive treatment is a clinical problem^2^. Even after surgical resection of these spinal cord gliomas, low grade spinal cord gliomas (WHO I and II) with a poor response to postoperative radiotherapy and chemotherapy, commonly reappeared after several months or several years. Given the highly variable and evolving nature of SCG, it is necessary to identify novel biomarkers that are specific for SCG and would help to improve the prediction of SCG^3^.

MicroRNAs (miRNAs) are small non-coding RNA molecules that bind to the 3′ untranslated region of a target mRNA^4^. miRNAs can either act as tumour suppressors or as oncogenes depending on their target gene^5–7^. By regulating the expression of the target genes, miRNAs play important roles in the development of cancers^8^. In addition, studies have shown that a single miRNA can affect the expression of multiple target genes^9,10^. miRNAs can be used as tumour biomarkers for prediction, diagnosis and prognosis^11^. For example, dysregulated miRNAs play an important role in the development and progression of gliomas^12,13^. However, the expression patterns and mechanisms of miRNAs in the SCG remain largely unknown.

Therefore, comprehensive analysis of the differentially expressed profiles of miRNAs and mRNAs in tumour tissue and circulating blood of patients with low or high grade SCG will help reveal the role of differential expression miRNAs and their target genes involved in SCG, and contribute to the elucidation of the pathogenesis of SCG and further benefit the diagnosis and staging treatment of patients.

## Results

### Spinal cord gliomas (SCG) Patients characteristic

**Figure 1:**
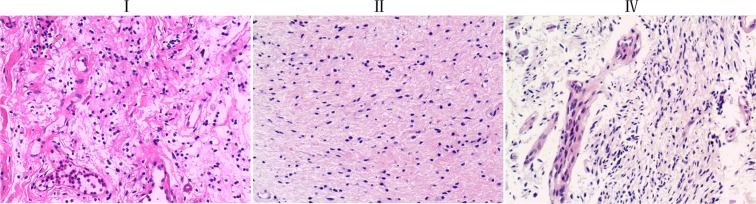
HE staining of tissue of patients with different grades of SCG. I The bipolar cells are partially arranged around the blood vessels and the Rosenthal’s fibers are visible. II Tumor cells are heteromorphic with deep nuclear staining, and tumor cell density is moderately low. IV The nuclear division of the cell is active, with obvious microvascular proliferation and obvious cell atypia.

We selected 10 patients with SCG who went through surgical treatment without preoperative chemotherapy or radiotherapy. According to WHO (2016) on the classification of neurological tumours^14^ and pathological section analysis of patient tumor tissue, the tissues and blood of 10 SCG patients were divided into high-grade (WHO IV) and low-grade (WHO II and WHO I), respectively. All the available clinical and follow-up data are included in Table 1 and Fig. 1.

**Table 1:** Clinical features of SC-GBM and L-SCG patients.

Patient ID	Age	Gender	Tumor pathology classification	Tumor grade	Tumor location	survival months
25343	28	Female	Glioblastoma	IV	T10-L1	Paraplegia*
26600	15	male	Glioblastoma	IV	C5-7	5
260002	37	male	Glioblastoma	IV	T1-5	3
28553	14	Female	Glioblastoma	IV	T1-8	Relapsed*
28378	11	male	Glioblastoma	IV	T9-12	Relapsed*
26051	48	male	Astrocytoma	II	C1-3	*
506353	27	Female	Astrocytoma	II	T5-7	>6
511691	45	Female	Astrocytoma	II	C6-T1	>8
27110	17	male	Pilocytic astrocytoma	I	T1-2	>12
503550	13	male	Pilocytic astrocytoma	I	T1-4	>15

SC-GBM = spinal cord glioblastoma, L-SCG = Low-grad Spinal cord gliomas, Patient ID = patient identification number, C = Cervical, L = Lumbar, T = Thoracic. *losing contact.

### DEmiRNAs (Differentially expressed miRNAs) in blood and tissue between SC-GBM and L-SCG

**Figure 2:**
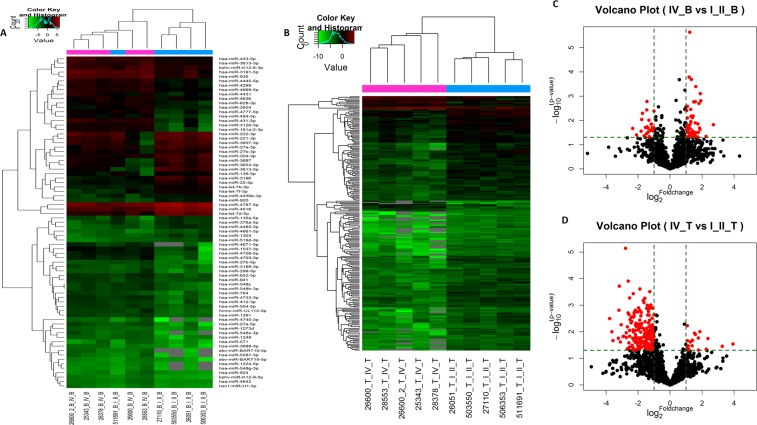
Differential expression of miRNAs in tissues and arterial blood between SC-GBM and L-SCG. Hierarchical clustering analysis the DEmiRNAs between SC-GBM and L-SCG blood (**A**) tissues (**C**) (fold change >2; P < 0.05; N = 5). Volcano plots. The vertical lines correspond to 2.0-fold up- and down-regulation between SC-GBM and L-SCG blood (**B**) and tissues (**D**), and the horizontal line represents a P-value. The red point represents the DEmiRNAs with statistical significance.

A total of 74 miRNAs were identified as DEmiRNAs between SC-GBM and low-grade spinal cord glioma (L-SCG) patient peripheral blood cells. Compared to the low-grade group, there were 49 up-regulated and 25 down-regulated miRNAs in the SC-GBM group (P < 0.05, fold change >2). Simultaneously, 207 DEmiRNAs were detected between SC-GBM and L-SCG patient tissue, of which 20 were upregulated and 187 were downregulated in SC-GBM group (P < 0.05, fold change >2). The microarray results clearly revealed the number of DEmiRNAs in tissue samples to be significantly higher than that in blood samples. Here, we list the first 10 differentially expressed (either upregulated or downregulated) miRNAs, respectively (Tables 2–5). A hierarchical clustering analysis and volcano plot were used for visualizing DEmiRNAs between the two groups based on their expression levels between samples. As shown in Fig. 2, these miRNAs displayed a general difference between SC-GBM and L-SCG patients.

**Table 2:** The top 20 up-regulated miRNAs in blood between SC-GBM and L-SCG.

Name	Fold change	P-value
hsa-miR-4740-3p	6.525656392	0.015113332
hsa-miR-5587-3p	4.736648534	0.045813524
hsa-miR-27a-5p	3.782315977	0.001552331
hsa-miR-3688-3p	3.68823972	0.00078339
hsa-miR-4703-3p	3.65325004	0.023568124
hsa-miR-431-5p	3.581687066	0.02074083
hsa-miR-3126-3p	3.508016612	0.00199045
hsa-miR-571	3.394631406	0.031366111
hsa-miR-181a-2-3p	3.335763967	0.013981023
ebv-miR-BART10-5p	3.311469302	0.048013163
hsa-miR-548o-3p	3.247652077	0.04832375
kshv-miR-K12-9-5p	3.036833321	0.000402608
hsa-miR-4671-5p	3.008413118	0.031651186
hsa-miR-4431	3.00071241	0.032710473
hsa-miR-4733-3p	2.992158897	0.001673458
hsa-miR-4708-5p	2.963448331	0.01269584
hsa-miR-299-5p	2.936149543	0.042478936
hsa-miR-412-3p	2.779311988	0.001726679
hsa-miR-1537-3p	2.767530197	0.033309459
ebv-miR-BART19-5p	2.709788765	0.006542985

**Table 3:** The top 20 down-regulated miRNAs in blood between SC-GBM and L-SCG.

Name	Fold change	P-value
hsa-miR-3196	0.198090894	0.021246601
hsa-miR-3613-5p	0.234047001	0.039956559
hsa-let-7d-5p	0.285012754	0.027815875
hsa-miR-4485-3p	0.293230214	0.009221795
hsa-miR-221-3p	0.302537514	0.005134891
hsa-miR-3687	0.324575439	0.043570637
hsa-miR-4516	0.342281362	0.01976555
hsa-miR-3653-3p	0.360303476	0.035669733
hsa-miR-376a-3p	0.364991188	0.00372444
hsa-miR-920	0.365749338	0.001653505
hsa-miR-1303	0.371727406	0.019028421
hsa-miR-3607-3p	0.371804803	0.018395651
hsa-miR-25-5p	0.381840394	0.02072343
hsa-let-7f-5p	0.382337557	0.030940673
hsa-miR-135a-5p	0.39990696	0.009806781
hsa-miR-204-3p	0.40780491	0.0470677
hsa-miR-4787-5p	0.415170289	0.048932902
hsa-miR-27a-3p	0.422492902	0.026147403
hsa-let-7b-3p	0.440087254	0.014552425
hsa-miR-222-3p	0.444593232	0.026170249

**Table 4:** Up-regulated miRNAs in tissues between SC-GBM and L-SCG.

Name	Fold change	P-value
hsa-miR-196b-5p	15.24663025	0.028931546
hsa-miR-301b-3p	9.562320427	0.034866815
hsa-miRPlus-A1083	5.134128878	0.017853778
hsa-miR-542-5p	4.78668274	0.045083866
hsa-miR-99a-5p	3.621954438	0.009847628
hsv2-miR-H10	3.412037878	0.048603892
hsa-miR-1469	3.397564668	0.017093603
hsa-miR-25-3p	2.985610133	0.012606392
hsa-miR-3940-5p	2.87457823	0.011321879
hsa-miR-1273f	2.642233043	0.021242596
hsa-miR-148a-3p	2.617968064	0.020746879
hsa-miR-199b-5p	2.597233508	0.033250216
hsa-miR-3195	2.401192179	0.024099904
hsa-miR-4708-3p	2.265626477	0.047564702
hsa-let-7c-5p	2.203631519	0.034172973
hsa-miR-92a-3p	2.18672811	0.033922439
hsa-miR-320b	2.12743175	0.041105671
hsa-miR-1268b	2.112420964	0.005909261
hsa-miR-4787-3p	2.109458023	0.021351637
hsa-miR-4739	2.069906337	0.04422456

**Table 5:** The top 20 down-regulated miRNAs in tissues between SC-GBM and L-SCG.

Name	Fold change	P-value
hsa-miR-302f	0.072919762	0.003163643
hsa-miR-4752	0.080358253	0.009939258
hsa-miR-196b-3p	0.084478269	0.021473288
hsa-miR-520g-3p	0.106302596	0.017467378
hsa-miR-212-5p	0.10874807	0.001550933
hsa-miR-4680-3p	0.111068791	0.00019238
hsa-miR-4724-3p	0.115814196	0.023906092
hsa-miR-658	0.119940191	0.005442459
hsa-miR-4477b	0.130600471	0.015040548
hsa-miR-1279	0.133656385	0.014645975
hsa-miR-4490	0.14550809	7.26109E-06
hsa-miR-4477a	0.149854027	0.010815332
hsa-miR-3144-5p	0.15021497	0.01620099
hsa-miR-5009-3p	0.156463316	0.043853059
hsa-miR-5009-5p	0.157194486	0.006468425
hsa-miR-3145-3p	0.161776923	0.001953459
hsa-miR-325	0.162684967	0.000123545
hsa-miR-506-3p	0.167906835	0.026082657
hsa-miR-3119	0.167976858	0.002214012
hsa-miR-3664-3p	0.171376881	0.038077497

**Figure 3:**
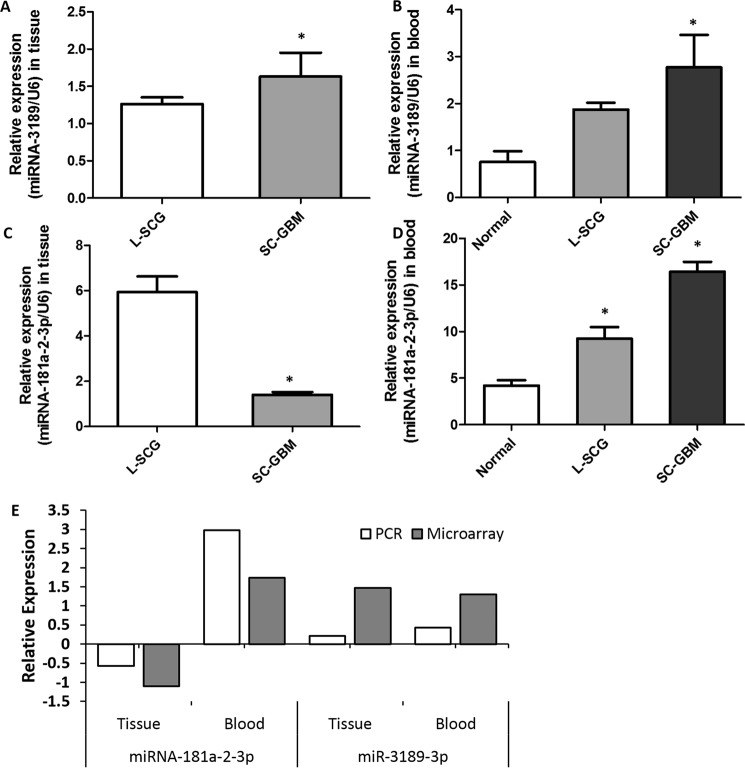
Validation of microarray data by qRT-PCR. Comparison of miR-181a-2-3p qRT-PCR data between groups in tissue (**A**) and blood (**B**). Comparison of miR-3189-3p qRT-PCR data between groups in tissue (**C**) and blood (**D**). (**E**) Comparison of the results of qRT-PCR and microarray for miR-181a-2-3p and miR-3189-3p. Results obtained with these two methods were consistent with each other. *P < 0.05 vs. L-SCG or Normal, n = 6.

To validate the results of the microarray, we randomly selected two differentially expressed miRNAs for qRT-PCR, miR-3189-3p were up-regulated both in tissue and blood in SC-GBM, and miR-181a-2-3p was down-regulated in tissue while being up-regulated in blood. The results of qRT-PCR were consistent with the microarray data and difference in RNA expression was statistically significant (P < 0.05) (Fig. 3). All data for the DEmiRNAs are provided in Supplementary Data 1.

### Prognostic indicators for disease relapse

**Figure 4:**
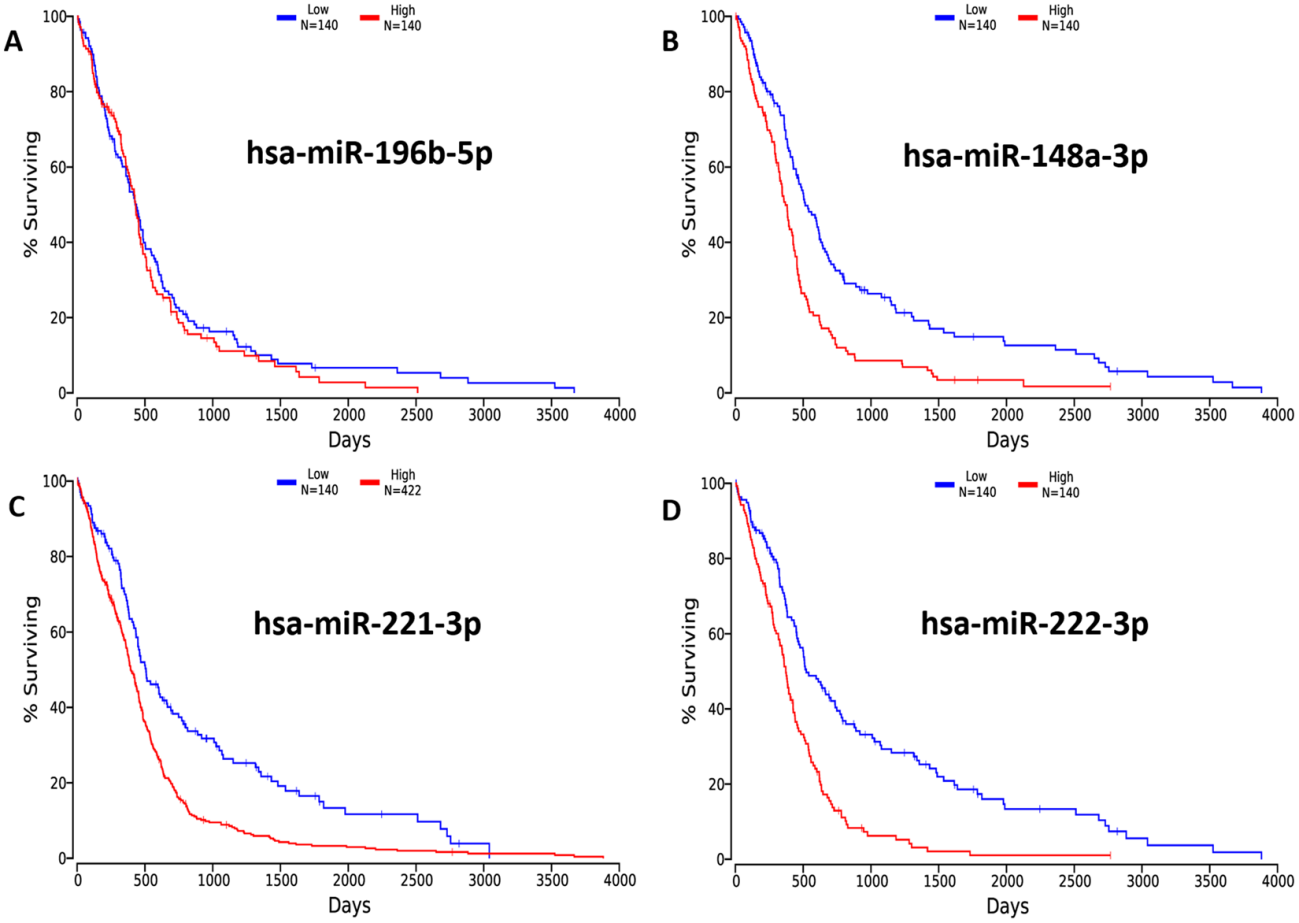
A Kaplan–Meier curve estimates the association of miRNAs and the survival of patients with GBM. (**A**) Association between the expression of has-miR-196b-5p and survival days in GBM patient tissue samples. (**B**) Association between the expression of has-miR-148a-3p and survival days in GBM patient tissue samples. (**C**) Association between the expression of has-miR-221-3p and survival days in GBM patient blood samples. (**D**) Association between the expression of has-miR-222-3p and survival days in both GBM patient blood and tissue samples.

To further investigate the role of the miRNA profile, we evaluated the GBM-related data available in the TCGA database using OncoLnc. Four differentially expressed DEmiRNAs (has-miR-196b-5p, has-miR-148a-3p, has-miR-221-3p, and has-miR-222-3p) in our study were identified in the TCGA database. We then performed an analysis to determine whether these four dysfunctional miRNAs are associated with cancer recurrence. We used the OncoLnc tool to link TCGA survival data to miRNAs. All patients included in the study were diagnosed with GBM. We observed that patients with lower expression of has-miR-196b-5p had longer survival (Fig. 4A), which confirmed our above microarray results that has-miR-196b-5p has a higher expression in high-grade glioma patients compared to patients with low-grade gliomas. Similarly, our microarray results revealed that has-miR-148a-3p was up-regulated in the high-level group compared to the low-level group, which was consistent to the survival analysis results that patients with low expression of has-miR-148a-3p had a longer lifespan (Fig. 4B). In addition, similar results were observed upon independent validation of has-miR-221-3p and has-miR-222-3p levels (Fig. 4C,D). These data further demonstrate differential expression of has-miR-196b-5p, has-miR-148a-3p, and has-miR-221-3p, and their expression pattern may be prognostic biomarkers for GBM patients.

### DEmiRNAs target prediction in SCG blood and tissue samples

**Figure 5:**
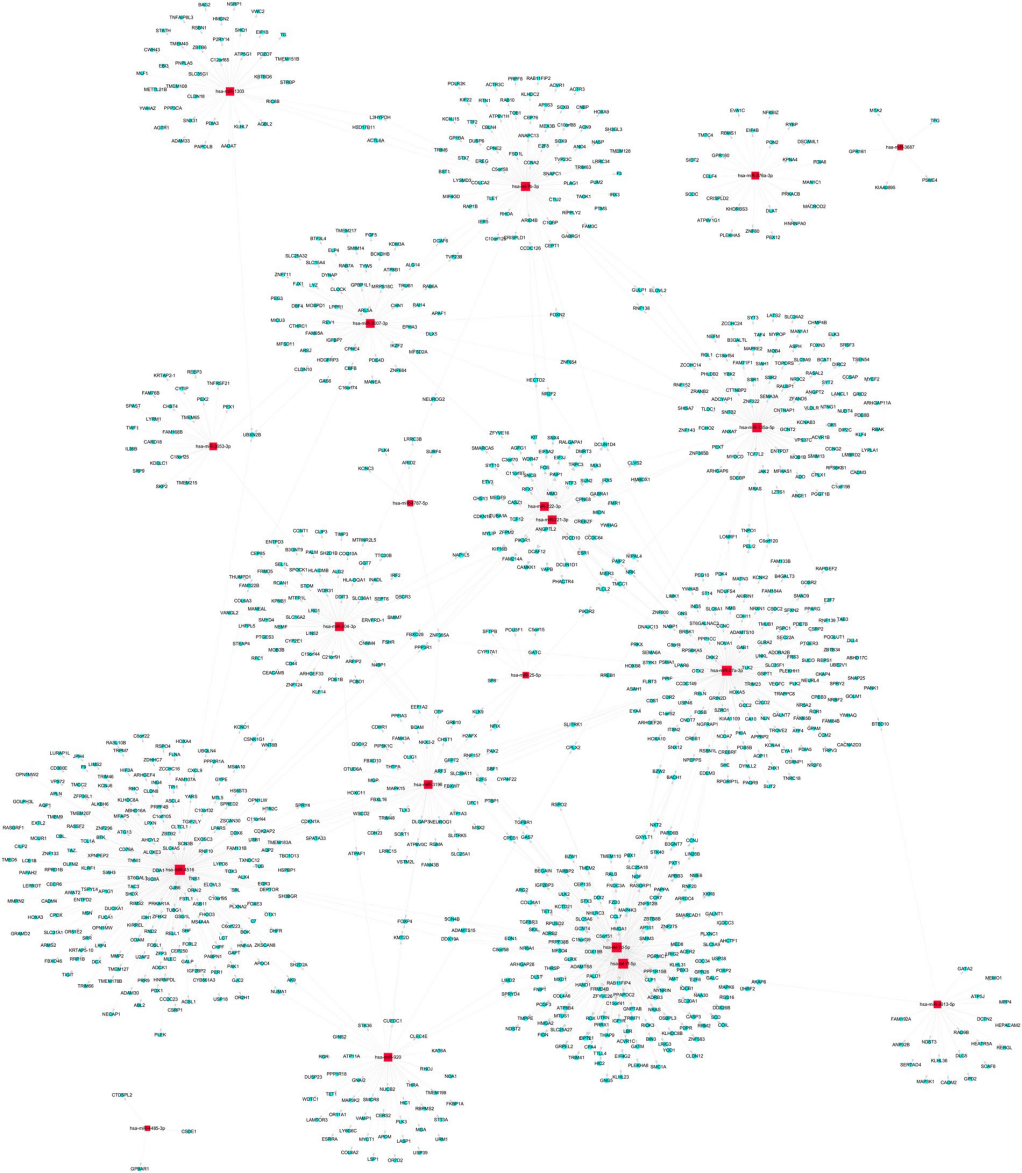
Network of differentially expressed MiRNA-mRNA in blood. Red-square nodes and Blue-round nodes are represented down-regulated miRNAs and mRNAs, respectively, and the solid line between the two nodes represents a correlation.

**Figure 6:**
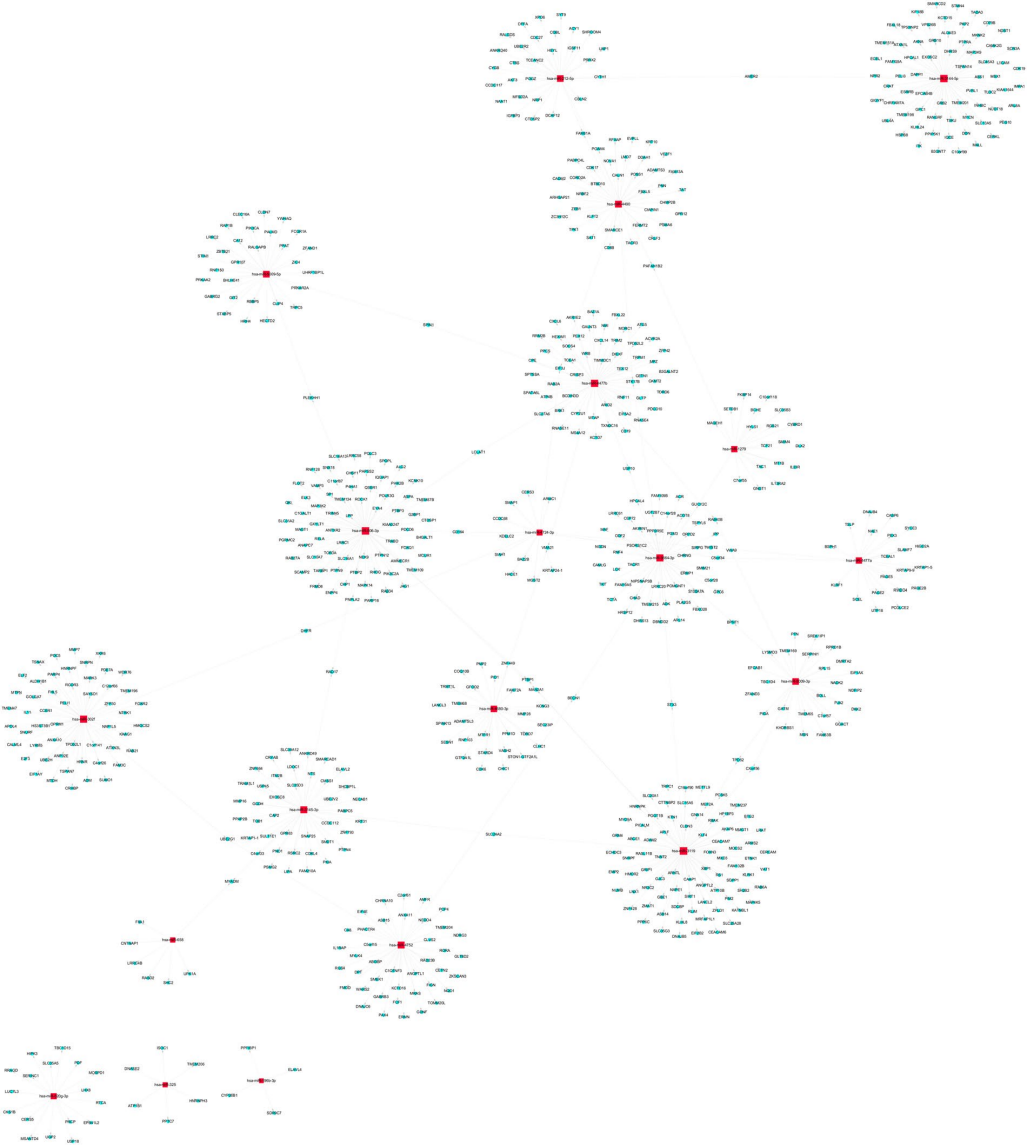
Network of differentially expressed MiRNA-mRNA in tissue. Red-square nodes and Blue-round nodes are represented down-regulated miRNAs and mRNAs, respectively, and the solid line between the two nodes represents a correlation.

In order to explore the potential regulatory effect of DEmiRNA on mRNAs, we also predicted target genes for DEmiRNAs. We selected the top 20 miRNAs to predict their target genes based on the fold change value of DEmiRNAs. By software-based prediction, there were 1468 and 728 target genes for upregulated and downregulated miRNAs in the tissue samples, respectively. In addition, 388 and 1378 target genes were found in blood samples of upregulated and downregulated miRNAs. Here we list the network diagram of downregulated miRNAs and their target genes in tissue and blood, respectively (Figs 5 and 6).

### GO analysis DEmiRNAs in SCG blood and tissue samples

**Figure 7:**
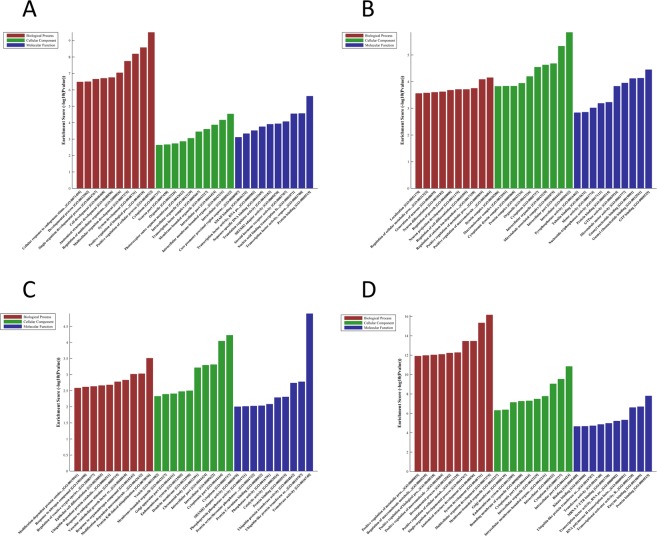
Top 10 enrichment (−log10 (P value)) of GO terms for DEmiRNAs in blood (**A**, downregulated and **B**, upregulated) and tissue (**C**, downregulated and **D**, upregulated) from the SC-GBM patients. The abscissa represents the GO term, from left to right in turn for the biological process, cell composition, molecular function, and the ordinate represents the enrichment factor of the gene that is annotated to the term.

We used the gene function analysis website (http://www.geneontology.org) to perform the Biological Process (BP), Molecular Function (MF), and Cellular Component (CC) analysis of DEmiRNAs with those are upregulated and downregulated in the SCG tissues and blood (Fig. 7; Supplementary Data 2). GO enrichment analysis in blood sample showed that up-regulated miRNA was enriched in 482 BP, 91 CC and 79 MF, while down-regulated mRNA was enriched in 813 BP, 59 CC and 109 MF. The most highly enriched up-regulated GO terms were “positive regulation of macromolecule metabolic process (BP)”, “intracellular (CC)”, and “GTP binding (MF)”, while down-regulated GO terms were most highly enriched in “positive regulation of cellular process (BP)”, “intracellular (CC)”, and “protein binding (MF)”. In the tissue sample, the up-regulated miRNA was enriched in 1239 BP, 162 CC and 164 MF, while down-regulated mRNA was enriched in 197 BP, 61 CC and 50 MF. The most highly enriched up-regulated GO terms were “multicellular organism development (BP)”, “intracellular part (CC)”, and “protein binding (MF)”, while down-regulated GO terms were most highly enriched in “protein K48-linked ubiquitination (BP)”, “cytoplasm (CC)”, and “transferase activity (MF)”. Our results suggest that miRNAs in both blood and tissue samples are associated with cell biological development-related items.

### Pathway analysis of DEmiRNAs in SCG blood and tissue samples

**Figure 8:**
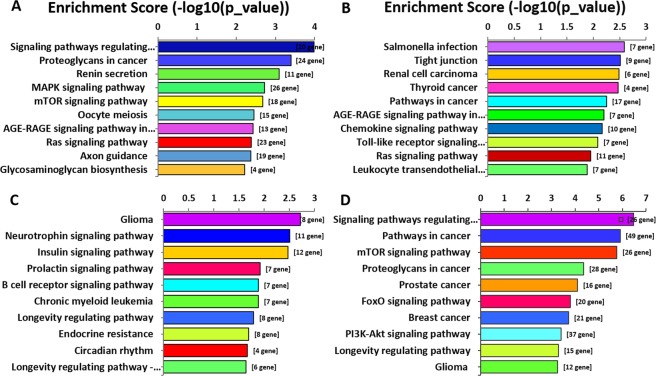
KEGG pathways enrichment analysis of SCG. Top 10 enrichment (−log10 (P value)) of pathways for DEmRNAs in blood (**A**, down-regulated and **B**, up-regulated) and tissue (**C**, down-regulated and **D**, up-regulated).

Pathway knowledge can provide disease marker information that is crucial to diagnosis, choice of drug, and patient treatment^15^. Based on the KEGG (Kyoto Encyclopedia of Genes and Genomes) database, the significance levels of DEgene pathways were analysed in our research (Fig. 8). The results showed that the down-regulated miRNAs in the blood was mainly involved in the “Signalling pathways regulating pluripotency of stem cells” pathway, and the up-regulated genes were mainly involved in the “salmonella infection” pathway. Furthermore, the differential expression genes in the tissue are mainly involved in “Glioma”, “Signalling pathways regulating pluripotency of stem cells” and “Pathways in cancer” pathways. KEGG pathway analysis indicated that miRNAs that are dysregulated in tissue and blood samples through the “Glioma”, “Signalling pathways regulating pluripotency of stem cells” and “Pathways in cancer” pathways, thereby affecting the progression of SCG disease.

### The common DEmiRNAs of tissue and blood samples in SC-GBM and L-SCG

In order to study the differences in miRNA expression between SCG tissue and blood, we compared the DEmiRNAs data in SCG tissue and blood and picked out the DEmiRNAs which were not only present in tissues but also in blood. We found that 9 up-regulated and 17 down-regulated miRNAs were simultaneously present in the blood and tissues of SC-GMB patients (Table 6). Interestingly, we also observed three DEmiRNAs, *hsa-miR-181a-2-3p, hsa-miR-764* and *kshv-miR-K12-6-3p*, whose expression in the tissue and blood were entirely opposite, i.e. they were all significantly up-regulated in the blood while being down-regulated in the tissue.

**Table 6:** Co-expression of DEmiRNAs between blood and tissue.

ID	Name	Blood		Tissue	
		FC	P-value	FC	P-value
**Up-regulated**					
46258	hsa-miR-1184	2.2546	0.0969	2.6751	0.4408
42702	hsa-miR-30c-1-3p	2.0758	0.2024	2.3802	0.3339
147731	hsa-miR-3189-3p	2.4706	0.0068	2.7780	0.1861
147679	hsa-miR-3197	2.2278	0.2495	3.1664	0.2333
169378	hsa-miR-4430	3.7769	0.1252	2.2844	0.2175
168928	hsa-miR-4431	3.0007	0.0327	2.4578	0.4755
30493	hsa-miR-548a-5p	2.6957	0.3233	2.6742	0.0992
145933	hsa-miR-652-5p	2.0440	0.0039	3.6343	0.2335
148673	hsv1-miR-H15	4.8409	0.2213	3.4020	0.2077
**Down-regulated**					
42769	hsa-let-7b-3p	0.4401	0.0146	0.2541	0.3962
5250	hsa-miR-105-5p	0.3436	0.2065	0.4136	0.0124
46404	hsa-miR-1244	0.4372	0.2428	0.2266	0.0543
148227	hsa-miR-1251-3p	0.4507	0.3436	0.3266	0.0085
145972	hsa-miR-141-5p	0.3104	0.2530	0.3995	0.0582
11022	hsa-miR-221-3p	0.3025	0.0051	0.4126	0.4049
11023	hsa-miR-222-3p	0.4446	0.0262	0.2693	0.2650
148215	hsa-miR-3591-3p	0.4094	0.2951	0.2117	0.0019
148430	hsa-miR-374c-5p	0.4851	0.1413	0.1743	0.0147
148499	hsa-miR-3910	0.4852	0.4121	0.2893	0.0931
169072	hsa-miR-3925-3p	0.3670	0.2833	0.3706	0.1074
169247	hsa-miR-4477a	0.4285	0.3173	0.1499	0.0108
168801	hsa-miR-4477b	0.4105	0.3063	0.1306	0.0150
169109	hsa-miR-5003-5p	0.4194	0.4575	0.2731	0.0197
42959	hsa-miR-514a-3p	0.3896	0.4511	0.3190	0.0154
17612	hsa-miR-555	0.4927	0.3098	0.3010	0.3019
168669	hsa-miR-5693	0.3598	0.2693	0.2233	0.0507

## Discussion

MiRNAs are involved in a variety of tumour development, including liver cancer, lung cancer, breast cancer, brain tumours and colon cancer^16,17^. Meanwhile, the dysregulated miRNAs also play a vital function in the occurrence and development of gliomas^18^. Some miRNAs are known to promote brain gliomas formation and progression by regulating the definition of cellular metabolism, invasion, and angiogenesis in glioma^19^. Their small size, stability, and targeting ability of various oncogenes have simultaneously identified miRNAs as candidates for biomarkers of brain gliomas. Several clinical studies have shown that surgical resection of SCG has far less effect than that of brain gliomas^20^. As a glioma of the central nervous system (CNS), SCG may also present a unique dysregulation pattern of miRNAs^21^. The disclosure of miRNA properties of SCG may also be beneficial to the diagnosis, prognosis and treatment of the tumour patients.

The expression of miRNAs in blood and tissues have tumour-related and tissue-specific features, and their expression is remarkably stable^22^. In order to get a more comprehensive data of miRNAs in SCG patients, we studied the expression profiles of miRNAs both in tissues and blood of SCG patients with different pathological grades using miRNA microarray. A total of 49 up-regulated miRNAs and 25 down-regulated miRNAs were identified in peripheral blood. At the same time, 20 up-regulated and 187 down-regulated miRNAs were observed in the spinal cord glioblastoma (SC-GBM) tissues compared to L-SCG (P < 0.05, FC > 2). A greater number of miRNAs were up-regulated in the SC-GBM group in blood samples, whereas in tissue sample, a greater proportion of miRNAs were down-regulated. We found that 9 up-regulated and 17 down-regulated miRNAs were consistently present in the blood and tissues of SC-GMB patients. Furthermore, we noticed that there are a few differentially expressed miRNAs (DEmiRNAs) whose expression levels starkly contrasted between tissue and blood samples. We speculate that this result may be due to the distinct functions of miRNAs present in spinal cord glioma tissues and blood. For example, three kinds of DEmiRNAs, hsa-miR-181a-2-3p, hsa-miR-764, and kshv-miR-K12-6-3p, which were up-regulated in blood but down-regulated in tissues. Previous research has approved that hsa-miR-764 is a biomarker for the diagnosis of hepatocellular carcinoma and lung adenocarcinoma^23,24^. This interesting result suggests us to further consider and inquire into the exact mechanism of hsa-miR-764 in the onset and progression of of SCG. In addition, overexpression of miR-K12-6-3p contributes to the spread of malignant tumours and angiogenesis and requires to deeper investigation^25^. Subsequently, we verified the differentially expressed miRNA-181a-2-3p and miRNA-3189-3p by RT-PCR and the results were consistent with the miRNA microarray, indicating that the consequence was reliable.

A previously reported study suggested miR-3196 to be a target of the tumour suppressor protein H2AX, and that the combination of its promoter and phosphorylated H2AX (γH2AX) can promote cancer cell apoptosis^26^. In SC-GBM blood samples, the expression of hsa-miR-3196 is significantly decreased, which suggested that it may act as a key factor in the development of SCG. At the same time, hsa-miR-4740-3p was remarkably upregulated in SC-GBM blood (FD = 6.53; P = 0.015). The function of hsa-miR-4740-3p is not known yet. However, the high expression in SC-GBM blood samples warrants further attention. Additionally, another down-regulated miRNA in the blood, hsa-miR-27a-3p, (FD = 0.42; P = 0.026), has been reported to serve as a prognostic and therapeutic target for GBM in the brain and its expression level can be used to identify grade II, III, and IV astrocytomas^27^. Therefore, we can also explore whether hsa-miR-27a-3p can be used as a target of SCG grading in various grades of SCG in the future studies.

B-cell translocation gene 2 (BTG2) is a direct functional target of miR-27a-3p in gastric cancer. Inhibition of miR-27a-3p significantly up-regulates BTG2 expression^28^. In order to further elucidate the mechanism and characteristics of DEmiRNAs in SCG, we constructed the miRNA-mRNA network. Interestingly, we observed that BTG2 is not only target the expression of hsa-miR-27a-3p in the blood but also regulates the expression of hsa-miR-506-3p, hsa-miR-3119 and hsa-miR- 212-5p in the tissue. This regulatory type prompts us that these four miRNAs may act as potential makers of diagnosis, treatment, and prognosis for SCG patients.

In tissue samples, the most significantly up-regulated miRNA was hsa-miR-196b-5p. Previous studies have reported the overexpression of hsa-miR-196b-5p to be associated with Ebola virus^29^, while low expression of it causes relapse in patients with multiple sclerosis^30^. This indicates that the dysregulation of hsa-miR-196b-5p plays an important role in regulating physiological function as well as pathology. In addition, miR-302f is most significantly down-regulated in SC-GBM patient tissues in this study, whereas it is up-regulated in gastric cancer tissues, confirming the tissue specificity of miRNAs^31^. Growth differentiation factor 15 (GDF15), has been shown to be one of the target genes for p53^32^. In this study, we found that GDF15 can directly target hsa-miR-3664-3p, a miRNA whose expression was downregulated in SC-GBM tissue (FD = 0.17; P = 0.038). It is well-established that miRNAs inhibit the expression of their target genes^33^. Therefore, the decrease of hsa-miR-3664-3p expression in SC-GBM may result in the increase of its target gene GDF15 and further increase the expression of p53. In addition, another GDF15-related gene miR-3189, consistent with previous findings^34^, was elevated in SC-GBM compared to L-SCG.

Pathway analysis identified several pathways related to cancer. For example, “Glioma” and “Pathways in cancer” are two of the most enriched pathways. In addition, “mTOR signalling pathway”, “Pathways in cancer”, “Proteoglycans in cancer” and “Signalling pathways regulating pluripotency of stem cells” are present in both tissue and blood DEmiRNAs, which have been shown to be involved in the pathogenesis of glioma^35,36^. It is interesting that the most significantly increased pathway in blood samples, “Salmonella infection” (*has-05132*), has been shown to inhibit the growth of glioma^37^. Consistent with these outcomes, our results also indicated that Salmonella infection pathway may play a crucial role in the self-destruction of SCG via genetic modification.

To the best of our knowledge, this is the first study to investigate the role of miRNAs in SCG pathology. We used miRNA microarray and bioinformatics to screen out differentially expressed miRNAs in the tissue and blood of gliomas of different grades, and found that the miRNA expression pattern between blood and cancer tissue samples only partially overlaps, probably due to the presence of immune cells or metastatic regions from other tumours in the blood circulatory system^38^. The evidence presented in this study provides a basis for further study of the regulatory role of miRNAs in the molecular mechanism of mRNAs. Future studies should also be undertaken to elucidate the functional relevance of miRNAs that have been shown in this study to be differentially expressed in disease.

## Materials and Methods

### Patients and samples preparation

In this study, based on the 2016 WHO Classification of the Central Nervous^14^, we selected 10 patients with SCG who went through surgical treatment without preoperative chemotherapy or radiotherapy in the department of neurosurgery of Capital Medical University Sanbo Brain Hospital (2016.02-2016.12). The study cohort including high-grade group (WHO IV, n = 5) and low-grade group (WHO I-II, n = 5) SCG patients; respectively, whichever arterial blood and tissue samples for miRNA analysis were collected during surgery. Subsequently, the patient’s clinical data, including survival data, was obtained by us. The research protocol was approved and supervised by The Ethics Committee of BUCM (Beijing University of Chinese Medicine) and Capital Medical University Sanbo Brain Hospital. All procedures performed in studies involving human participants were in accordance with the ethical standards of the institutional and with the 1964 Helsinki declaration and its later amendments or comparable ethical standards. All patients in this study gave written informed consent, including adults, whose informed consent came from the participants themselves; And the informed consent by parents of all minor participants (age below 18). Tissue samples were pathologically confirmed, and were stored in liquid nitrogen immediately after surgical resection. Blood samples were stored in EDTA tubes and placed in liquid nitrogen for subsequent use.

### Tumor histopathology

We used Hematoxylin-eosin (HE) staining to assess the grade of SCG tissue samples. Briefly, tumor tissue were collected from different patients, and first fixed in 4% neutral formaldehyde fixative, then embded in paraffin. Sections of 3–5 mm thicknesses were used for HE staining, which was conducted according to convention methods. Finally, the morphological changes in tissue section was observed using an optical microscope (Olympus, Tokyo, Japan).

### Microarray determination

We used the 7th generation human miRNA array (containing 3100 capture probes), using tissue and circulating blood from five SC-GBM patients and five low grade SCG. Total miRNAs were hybridized and labelled following the manufacturer’s manual (Exiqon). Afterwards, an Axon GenePix 4000B microarray scanner (Axon Instruments, Foster City, CA) was used to scan the slides. Then, the scanned images were imported into GenePix Pro 6.0 software (Axon) for grid alignment and data extraction. After median normalization, significant DEmiRNAs between two groups were identified through FC and P-value.

### Quantitative Real-time PCR verification

RT-PCR was conducted according to An *et al*. protocol^39^. Total RNA was extracted from SCG tissue and blood with Trizol reagent (Invitrogen, Carlsbad, CA, USA). Complementary DNA (cDNA) was synthesized with a cDNA Reverse Transcription Kit (Invitrogen Life Technologies, USA) using 2 μg of total RNA, and miRNA expression was measured by quantitative PCR using SYBR Premix ExTaq and an MX3000 instrument. The primers and miRNA used in this study are listed in Table 7. PCR data were normalized to U6 to calculate miRNA-181a-2-3p and hsa-miR-3189-3p.

**Table 7:** MiRNA and mRNA primers for quantitative PCR analysis.

Primer name	Sequence
U6 (H)	F: 5′GCTTCGGCAGCACATATACTAAAAT3′ R: 5′CGCTTCACGAATTTGCGTGTCAT3′
hsa-miR-181a-2-3p	F: 5′ GCGCGACCACTGACCGTTGAC3′ R: 5′ ATCCAGTGCAGGGTCCGAGG3′
hsa-miR-3189-3p	F: 5′ CCGCGCCCTTGGGTCTGATG3′
	R: 5′ ATCCAGTGCAGGGTCCGAGG3′

### Link TCGA survival data to miRNA

We use the OncoLnc tool^40^ (http://www.oncolnc.org.) to link TCGA survival data for miRNAs. All study participants were diagnosed with GBM. First, select a single miRNA name (not case-sensitive). The Kaplan-Meier analysis option in the search results page was selected. We divided the patients into non-overlapping upper and lower sections, 25% upper and 25% lower. Finally, the drawn Kaplan-Meier diagram was displayed, and the CSV file with raw data used for the plot (Supplementary Data 3) was downloaded.

### Differential miRNA target gene prediction

MirdbV5 and TaregetScan7.1, target prediction databases, were used to predict and analyse the target gene of abnormal expression of miRNAs. Then the result of the prediction takes the overlapping part of the gene obtained by these two databases, which were used to GO and KEGG Pathway analysis.

### GO and pathway analysis

GO includes molecular, cellular and biological processes three sub-items. We used (http://www.geneontology.org) for GO analysis to obtain significantly enriched GO articles and corresponding genes to deduce important biological functions involved in DEmiRNAs. Meanwhile, based on the newest KEGG database, find out the pathways which are most associated with DEmiRNAs. Fisher’s exact test is used for identify pathway and GO entries which are significant correlated DEmiRNAs (p < 0.05).

### Statistical analysis

SPSS software (20.0 Version) was used for statistical analyses. All results are expressed as mean ± SEM. Comparisons between multiple groups were analysed using one-way ANOVA analysis. Multivariate Cox regressions were performed with the coxph function from the R survival library^40^. A probability value of P less than 0.05 was considered significant.

## Supplementary information

Supplementary data 1

Supplementary data 2

Supplementary data 3

## References

[1] Ma CC (2016). Long non-coding RNA ATB promotes glioma malignancy by negatively regulating miR-200a. J Exp Clin Cancer Res..

[2] Tobias A, Ahmed A, Moon KS, Lesniak MS (2013). The art of gene therapy for glioma: a review of the challenging road to the bedside. J Neurol Neurosurg Psychiatry..

[3] Duan R (2015). HOXA13 is a potential GBM diagnostic marker and promotes glioma invasion by activating the Wnt and TGF-beta pathways. Oncotarget..

[4] Baulina NM, Kulakova OG, Favorova OO (2016). MicroRNAs: The Role in Autoimmune Inflammation. Acta Naturae..

[5] Xue H (2016). MicroRNA-584-3p, a novel tumor suppressor and prognostic marker, reduces the migration and invasion of human glioma cells by targeting hypoxia-induced ROCK1. Oncotarget..

[6] Fan YH (2015). Overexpression of miR-98 inhibits cell invasion in glioma cell lines via downregulation of IKKepsilon. Eur Rev Med Pharmacol Sci..

[7] Chai C (2016). Circulating miR-199a-3p in plasma and its potential diagnostic and prognostic value in glioma. Eur Rev Med Pharmacol Sci..

[8] Liu Z (2017). MicroRNA-153 regulates glutamine metabolism in glioblastoma through targeting glutaminase. Tumour Biol..

[9] Mo FF (2017). Jiang Tang Xiao Ke Granule Play an Anti-diabetic Role in Diabetic Mice Pancreatic Tissue by Regulating the mRNAs and MicroRNAs Associated with PI3K-Akt Signaling Pathway. Front Pharmacol..

[10] Ben-Hamo R, Efroni S (2015). MicroRNA regulation of molecular pathways as a generic mechanism and as a core disease phenotype. Oncotarget..

[11] Reddy KB (2015). MicroRNA (miRNA) in cancer. Cancer Cell Int..

[12] Deng D (2016). MicroRNA-124-3p regulates cell proliferation, invasion, apoptosis, and bioenergetics by targeting PIM1 in astrocytoma. CANCER SCI..

[13] Song Hang, Zhang Yao, Liu Na, Zhao Sheng, Kong Yan, Yuan Liudi (2016). miR-92a-3p Exerts Various Effects in Glioma and Glioma Stem-Like Cells Specifically Targeting CDH1/β-Catenin and Notch-1/Akt Signaling Pathways. International Journal of Molecular Sciences.

[14] Louis DN (2016). The 2016 World Health Organization Classification of Tumors of the Central Nervous System: a summary [J]. Acta Neuropathologica.

[15] Jiang G (2017). Relationships of circular RNA with diabetes and depression. Sci Rep..

[16] Persson H (2011). Identification of new microRNAs in paired normal and tumor breast tissue suggests a dual role for the ERBB2/Her2 gene. Cancer Res..

[17] Mathupala SP, Guthikonda M, Sloan AE (2006). RNAi based approaches to the treatment of malignant glioma. Technol Cancer Res Treat..

[18] Ohnishi YI (2017). Promotion of astrocytoma cell invasion by micro RNA-22 targeting of tissue inhibitor of matrix metalloproteinase-2. J Neurosurg Spine..

[19] Beyer, S. *et al*. The Role of miRNAs in Angiogenesis, Invasion and Metabolism and Their Therapeutic Implications in Gliomas. *Cancers (Basel)*. **9**(7) (2017). 10.3390/cancers9070085 PMC5532621 28698530

[20] Hongyun H, Lin C (2013). The concept of “growth boundary of glioma” and total resection of brain and spinal gliomas.Chinese. Journal of Clinical Medicine.

[21] Drusco A (2015). A differentially expressed set of microRNAs in cerebro-spinal fluid (CSF) can diagnose CNS malignancies. Oncotarget..

[22] Molina-Pinelo S (2012). Association between the miRNA signatures in plasma and bronchoalveolar fluid in respiratory pathologies. Dis Markers..

[23] Chen Y, Chen J, Liu Y, Li S, Huang P (2015). Plasma miR-15b-5p, miR-338-5p, and miR-764 as Biomarkers for Hepatocellular Carcinoma. Med Sci Monit..

[24] Kim H (2017). MicroRNA expression profiles and clinicopathological implications in lung adenocarcinoma according to EGFR, KRAS, and ALK status. Oncotarget..

[25] Li W (2016). The SH3BGR/STAT3 Pathway Regulates Cell Migration and Angiogenesis Induced by a Gammaherpesvirus MicroRNA. Plos Pathog..

[26] Xu C, Zhang L, Duan L, Lu C (2016). MicroRNA-3196 is inhibited by H2AX phosphorylation and attenuates lung cancer cell apoptosis by downregulating PUMA. Oncotarget..

[27] Rivera-Diaz M (2015). MicroRNA-27a distinguishes glioblastoma multiforme from diffuse and anaplastic astrocytomas and has prognostic value. Am J Cancer Res..

[28] Zhou L (2016). MiR-27a-3p functions as an oncogene in gastric cancer by targeting BTG2. Oncotarget..

[29] Sheng M (2014). Hsa-miR-1246, hsa-miR-320a and hsa-miR-196b-5p inhibitors can reduce the cytotoxicity of Ebola virus glycoprotein *in vitro*. Sci China Life Sci..

[30] Selmaj I (2017). Global exosome transcriptome profiling reveals biomarkers for multiple sclerosis. Ann Neurol..

[31] Yao Y (2009). MicroRNA profiling of human gastric cancer. Mol Med Rep..

[32] Jones MF (2015). Growth differentiation factor-15 encodes a novel microRNA 3189 that functions as a potent regulator of cell death. Cell Death Differ..

[33] Zhang Y, Zhao S, Xu Z (2016). Network and pathway analysis of microRNAs, transcription factors, target genes and host genes in human glioma. Oncol Lett..

[34] Jeansonne D (2015). Anti-tumoral effects of miR-3189-3p in glioblastoma. J Biol Chem..

[35] Chao Y (2015). Mst1 regulates glioma cell proliferation via the AKT/mTOR signaling pathway. J Neurooncol..

[36] Liu M (2017). Phosphorylated mTOR and YAP serve as prognostic markers and therapeutic targets in gliomas. Lab Invest..

[37] Mehta N (2017). Bacterial Carriers for Glioblastoma Therapy. Mol Ther Oncolytics..

[38] Nagy, Z. B. *et al*. Comparison of Circulating miRNAs Expression Alterations in Matched Tissue and Plasma Samples During Colorectal Cancer Progression. *Pathol Oncol Res* (2017). 10.1007/s12253-017-0308-1 28980150

[39] Mo FF (2017). Jiang Tang Xiao Ke Granule Play an Anti-diabetic Role in Diabetic Mice Pancreatic Tissue by Regulating the mRNAs and MicroRNAs Associated with PI3K-Akt Signaling Pathway[J]. Frontiers in Pharmacology.

[40] Anaya Jordan (2016). OncoLnc: linking TCGA survival data to mRNAs, miRNAs, and lncRNAs. PeerJ Computer Science.

