# Publisher Correction: Comparison of Alterations in miRNA Expression in Matched Tissue and Blood Samples during Spinal Cord Glioma Progression

**DOI:** 10.1038/s41598-020-62065-0

**Published:** 2020-04-22

**Authors:** Tian An, Tao Fan, Xin Qing Zhang, Yu-Fei Liu, Jiangpinghao Huang, Cong Liang, Bo-Han Lv, Yin-Qian Wang, Xin-Gang Zhao, Jia-Xian Liu, Yu-Huan Fu, Guang-Jian Jiang

**Affiliations:** 1 Traditional Chinese Medicine School, Beijing University of Chinese Medicine, Beijing, 100029 China; 2 Department of Neurosurgery, Sanbo Brain Hospital, Capital Medical University, Beijing, 100093 China; 3 Department of Neurosurgery, ChuiYangLiu Hospital affiliated to Tsinghua University, Beijing, 100022 China; 4 The Third Affiliated Hospital, Beijing University of Chinese Medicine, Beijing, 100029 China; 5 University of Southern California, Los Angeles, CA 90007 USA; 6 Molecular Development and Diagnosis of Tumor Pathology, Department of Basic Medicine, Tangshan Vocational and Technical College, Tangshan, 063000 China

Correction to: *Scientific Reports* 10.1038/s41598-019-42364-x, published online 24 June 2019

In the original version of this Article, Tao Fan was omitted as a corresponding author. Correspondence and request for materials should also be addressed to fant@ccmu.edu.cn. This error has now been corrected in the HTML and PDF versions of the Article.

